# Effects of *Lactobacillus acidophilus* on gut microbiota composition in broilers challenged with *Clostridium perfringens*

**DOI:** 10.1371/journal.pone.0188634

**Published:** 2017-11-30

**Authors:** Zhui Li, Weiwei Wang, Dan Liu, Yuming Guo

**Affiliations:** State Key Laboratory of Animal Nutrition, College of Animal Science and Technology, China Agricultural University, Beijing, China; University of New England, AUSTRALIA

## Abstract

This study shows the effects of dietary supplementation with *Lactobacillus acidophilus* on the gut microbiota of broiler chickens challenged with *Clostridium perfringens* infection during a 21-day period according to pyrosequencing of the 16S ribosomal RNA gene. In a 2 × 2 factorial arrangement of treatments, 308 1-day-old male Arbor Acres broiler chicks were analyzed for the effects of the probiotic (groups without or with *L*. *acidophilus* supplementation), pathogen challenge (groups without or with *C*. *perfringens*), and the effects of interaction. The infection decreased the number of Observed species, Chao1, and ACE of ileal microbiota and increased Chao1 of cecal microbiota of broilers, whereas *L*. *acidophilus* supplementation decreased the Shannon index of the ileal microbiota. Shannon index and Simpson indices were lower in the ileal microbiota than in the cecal microbiota. In the ileal microbiota, the control group had higher relative abundance of *Lachnospiraceae* and *Ruminococcaceae* in comparison with the other groups; however, the relative abundance of *Gammaproteobacteria* was significantly higher in the challenge group than in the other groups. *C*. *perfringens* infection tended to increase lactate concentration and decreasedconcentrations of formate, acetate and propionate in the ileum; decreased isobutyrate concentration; and tended to decrease isovalerate concentration in the cecum. Besides, *L*. *acidophilus* supplementation increased the concentration of lactate and butyrate and decreased concentrations of formate and propionate in the ileum, and increased concentrations of lactate and valerate in the cecum. In conclusion, *C*. *perfringens* infection and/or dietary supplementation with *L*. *acidophilus* modulated the relative abundance of some bacteria taxa, and the *L*. *acidophilus* supplementation helped to restore the microbial community disrupted by *C*. *perfringens* infection.

## Introduction

Necrotic enteritis (NE) is a universal poultry disease, which is mainly caused by *Clostridium perfringens* type A [1]. Globally, it costs $ 2 billion per year to the poultry industry owing to the poor growth performance, mortality of the birds and prevention expenses [2]. With the withdrawal of antibiotic growth promoters from poultry feed, this infection tends to break out [3] and has been a threat to poultry welfare and human food security.

*Lactobacillus* has been widely used as probiotics in feed to improve poultry growth performance and overall health [4, 5]. Some studies have shown that *Lactobacillus acidophilus* can suppress intestinal pathogens [6, 7]. Our previous study revealed that the probiotic *L*. *acidophilus* (used in the present study) can ameliorate the inflammatory response, and inhibit the colonization of some pathogens. Nonetheless, far less is known about the *L*. *acidophilus*-mediated changes in the *C*. *perfringens* challenged chicken microbiota. In this study, we used high-throughput sequencing of the V3-V4 region of the 16S ribosomal RNA (rRNA) gene to assess the ileal and cecal microbiota of unchallenged or challenged chickens fed diets without or with *L*. *acidophilus* supplementatioin. We also measured the concentrations of lactate and short-chain fatty acids (SCFAs) in the ileum and cecum.

## Methods

### Birds, diet, and experimental design

The experiment was approved by the China Agricultural University Animal Care and Use Committee.

A total of 308 1-day-old male Arbor acres broilers were obtained from a local commercial hatchery and used in a 2 × 2 factorial arrangement of treatments to study the effects of a probiotic (groups without or with *L*. *acidophilus* supplementation), a pathogen challenge (groups without or with *C*. *perfringens*infection) and their interactive effects. All the birds were weighed and randomly assigned to four treatments [Control group (CTL), *L*. *acidophilus* supplementationgroup (LA), Challenge group (CLG), and Challenge group supplemented with *L*. *acidophilus* (LACLG)] with seven replicates per treatment. All the birds were offered free access to feed and water throughout the 21-d study period.

A corn—soybean meal basal diet in mash form was formulated to meet the nutrient requirements recommended by the feeding standard of China (NY/T 2004) for broilers (Additional file: S1 Table). The probiotic *L*. *acidophilus* (Synlac Material Technology Co., Ltd, Nanjing, China) was added to the basal diet at 40 mg/kg to provide 4 × 10^5^ colony-forming units (cfu)/(kg of feed). The probiotic was progressively diluted and mixed with basal diet homogeneously every 4 d.

### The *C*. *perfringens* challenge

The *C*. *perfringens* challenge method used in our experiment was developed by Dahiya et al. (2005) [8] and modified by Liu et al. (2010) [9]. Briefly, a chicken *C*. *perfringens* type A field strain (CVCC 2030) was isolated from a clinical case of NE, which was obtained from the China Veterinary Culture Collection Center (Beijing, China). The organism was cultured anaerobically on tryptose-sulfite-cycloserine for 18 h at 37°C and then aseptically inoculated into a cooked meat medium and incubated anaerobically for 8h at 37°C. The birds of challenged groups were orally gavaged once a day with actively growing culture of wild-type *C*. *perfringens* (2.0 × 10^8^ cfu/mL, 1.0 mL/bird) to cause gut damage on days 14–20, and the unchallenged birds were gavaged with the same volume of the sterilized cooked meat medium.

### Sample collection and DNA extraction

Five birds per treatment were randomly chosen from different replicates and chickens were rendered unconscious by intravenously injection of pentobarbital sodium (100 mg/[kg body weight]) just before slaughter by exsanguination; next, the digesta samples were collected in the ileum and cecum. The content of the terminal half of the ileum (defined as the region between Meckel’s diverticulum until the point 2-cm cranial to the ileo-cecal junction) and cecum were collected within 5 min of euthanasia, immediately placed in cryogenic vials, snap-frozen in liquid nitrogen, delivered to the laboratory and stored at −80°C until DNA extraction. Genomic DNA was isolated from ~200 mg of digesta from the ileum and caecum using a commercial kit (QIAamp DNA Stool Mini Kit, Qiagen Inc., Valencia, CA). The DNA concentration and purity were determined on a NanoDrop 2000 spectrophotometer (Thermo Scientific, MA, USA).

### Pyrosequencing

The purified genomic DNA at the normalized concentration (20 ng/uL) served as a template for analysis of the microbial communities. The V3-V4 region of the 16S rRNA gene was amplified with the universal eubacterial primers (341 F: 5′-CCTAYGGGRBGCASCAG-3′ and 806R: 5′-GGACTACNNGGGTATCTAAT-3′). Primer sequences were modified by adding Illumina adaptor A to the 5’ ends of the forward primers, and adaptor B followed by 12 nucleotide barcode sequences to the 3’ ends of the reverse primers. The 50 μL reaction system consists of 20 μL Premix Ex Taq (Takara Biotechnology, Dalian, China), 0.4 μL of each primer (10 μM), 4 μL of five-fold diluted template DNA (1–10 ng) and 25.2 μL of sterilized water. Thermal cycling conditions were as follows: initial denaturation of 3 min at 94°C; six touch down cycles of 45 s at 94°C, 60 s from 65 to 58°C, and 70 s at 72°C; followed by 22 cycles of 45 s at 94°C, 60 s at 58°C, and 60 s at 72°C, with final extension at 72°C for 10 min. The PCR products were purified with the Qiagen Gel Extraction Kit (Qiagen, Germany), and then sequenced on the HiSeq2500 platform (Illumina, San Diego, CA, USA) at Novogene, Beijing, China.

### Data processing

Raw sequences generated by HiSeq paired-end sequencing were merged by means of fast length adjustment of short reads (FLASH) [10] with Q30 of clean full-length reads ranging from 95.0% to 95.8%. Barcodes and primers were subjected to trimming, where maximal two-base differences in barcodes and no primer mismatches were permitted. Sequences were excluded if they did not meet the default QIIME quality criteria. Sequences with an average quality score less than 25 in a sliding window of 50 nucleotides were also discarded. The sequence data were denoised using the denoise_wrapper.py command in QIIME. Chimeras were identified by means of the Uparse software and removed. Then the Effective Tags were obtained and applied to further analyses.

Uparse was used to select operational taxonomic units (OTUs) at 97% similarity [11] on the Bio-Linux platform. Representative sequences were processed via the QIIME pipeline [12]. LCA alignment assignment was carried out based on the Silva database [13]. Resampling according to the minimal sequence numbers across all the samples was performed before the downstream analyses. Community composition provides the classification information at different taxonomic levels. Shifts in bacterial community composition were visualized byprincipal component analysis (PCA) based on the 97% OTU similarity across the four treatment groups.

### Ileal and cecal SCFA and lactate concentrations

A 0.5-g sample of ileal or cecal digesta was weighed into a 10-mL polypropylene tube, then 8 mL of deionized water was added, an ultrasonic bath was applied for 30min, and the mixture was centrifuged for 10 min at 8000 revolutions per minute. The resulting suspension was diluted 10-fold and passed through a 0.22 μm-filter. Next, 25 μL of the extracted sample solution was studiedon a high performance ion chromatography system (ICS-3000; Dionex, USA) and analyzed by conductivity detection. The organic acids were separated on an AS11 analytical column (250 × 4mm) and an AG11 guard column under the following gradient conditions (the gradient was based on potassium hydroxide): 0-5min, 0.8–1.5mM; 5–10 min, 1.5–2.5 mM; and 10-15min, 2.5mM; the flow rate was 1.0 mL/min. The units of measurement of lactate and SCFAs were μg/(g digesta).

### Statistical analyses

To compare the microbial community structures, a plot of PCA was constructed in QIIME. ANOSIM with 999 permutations was employed to detect statistical significance of difference between microbial communities in different groups. This test yields a value of R, normally on the scale from 0 to 1, which is based on the average rank similarity among groups and replicates within each group [14]. R = 0 indicates that two groups are similar whereas R = 1 shows perfect separation between groups. Differentially abundant taxa were identified by the linear discriminant anylysis (LDA) effect size (LEfSe) method [15]. The LEfSe algorithm involves the nonparametric factorial Kruskal-Wallis test (α = 0.05) to analyze differences between classes (treatments). To normalize sequencing depth, the lowest counts among samples were randomly subsampled in each library 1,000 times and average values were selected to measure diversity indices. To evaluate the α-diversity in samples, the number of observed OTUs, Shannon index, Simpson index, Chao1, and ACE were computed in QIIME. Differences between the mean values were identified by two-factorial analysis of variance and Duncan’s multiple-comparison test in the SPSS 20.0 software. The relation between bacterial genera and SCFA concentrations was evaluated by the Pearson correlation test. The differences in within-community (α) diversity indices of gut microbiota and in SCFA concentrations among the treatment groups were subjected to two-factorial analysis of variance in the SPSS 20.0 software, and each cage was considered an experimental unit.

## Results

### The quality of sequencing data

In total, 3,678,796 and 3,896,271 pyrosequencing reads were obtained from 20 ileal samples and 20 cecal samples, respectively. After quality was checked and chimeric sequences were removed, the average number of reads generated from the ileal samples per bird was 53,708 (±7,093 [standard deviation; SD]) and 56,017 (±11,769) for the cecal samples, with the median read length of 419 (± 6.08) base pairs and 411 (±3.42) base pairs, respectively. The estimate of Good’s coverage reached > 99.6% for all the ileal and cecal samples. Rare OTUs (< 0.005% of all OTUs) were removed, and then 1,229 OTUs were retained for subsequent analysis.

### Effects of the *C*. *perfringens* challenge and *L*. *acidophilus* supplementation on the ileal microbiota

**Fig 1:**
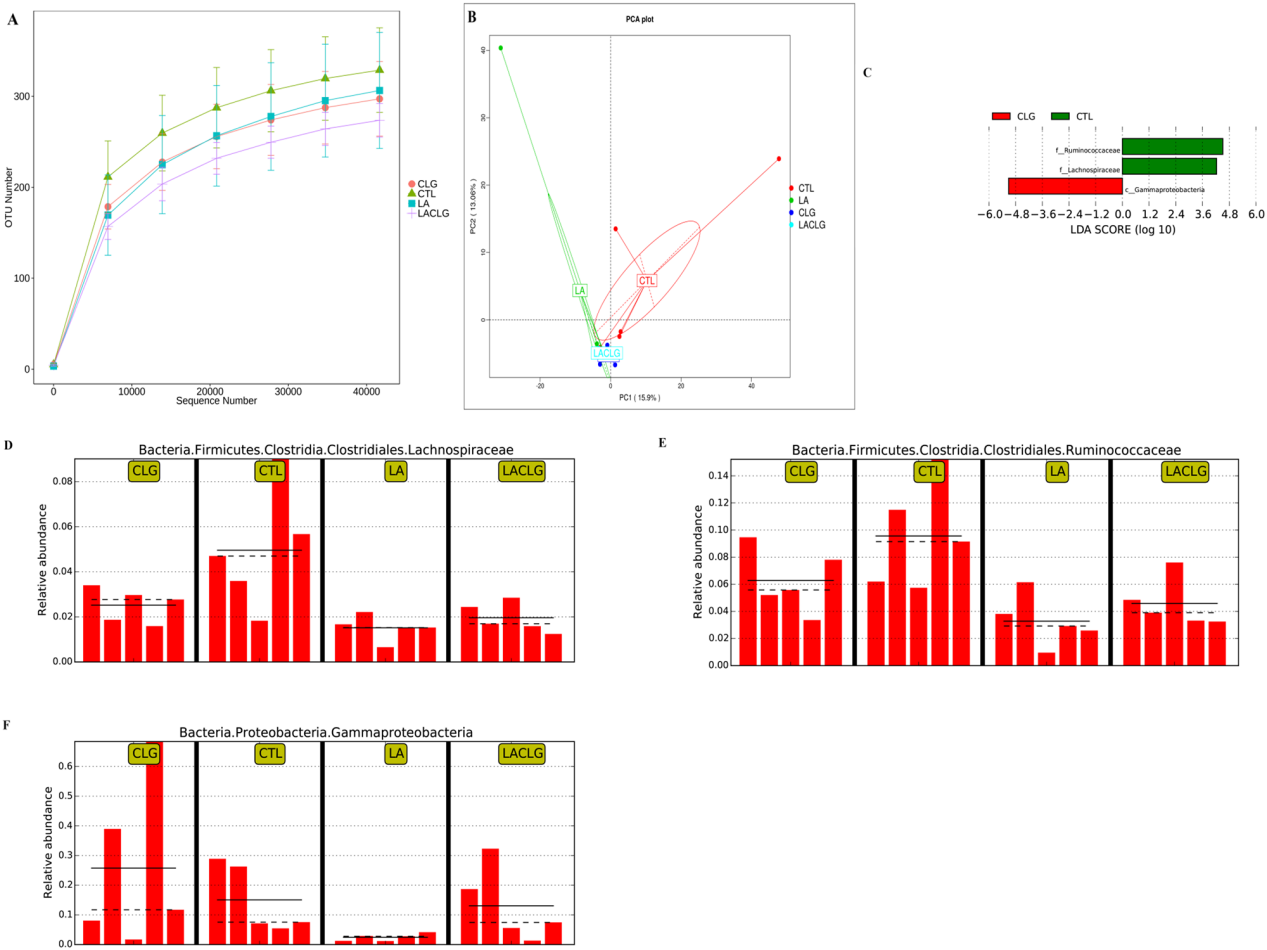
Diversity and composition of ileal microbiota. Rarefaction curves of the observed OTUs (**A**) for ileal samples. The community structure among the treatment groups did not differ according to the Principle component analysis (PCA) of 20 ileal samples (**B**). Linear discriminant analysis (LDA) effect size (LEfSe) showed the phylotypes that differ among treatment groups with statistical and biological significance (**C**). Histograms indicate the highest relative abundance of the families *Lachnospiraceae* (**D**) and *Ruminococcaceae* (**E**) in the ileal microbiota of the CTL group, and of the class *Gammaproteobacteria* (**F**) in the ileal microbiota of the CLG group. CTL: control group; LA: *L*. *acidophilus* supplementation group; CLG: Challenge group; LACLG: challenge group supplemented with *L*. *acidophilus*.

The number of observed species and Shannon, Simpson, Chao1 and ACE indices were calculated to assess α diversity (Table 1). According to the rarefaction curves for the observed OTUs (Fig 1A), the sequencing depth in the present study was sufficient to achieve high sampling coverage (99.9%) of all OTUs present in the ileal samples. The *C*. *perfringens* challenge decreased the number of observed species (*P* < 0.05), Chao 1 (*P* < 0.05), and ACE (*P* < 0.05) in the ileum. *L*. *acidophilus* addition to the diet decreased the ileal Shannon index (*P* < 0.05), irrespective of the *C*. *perfringens* challenge. By contrast, the Simpson index was not affected either by the challenge (*P* > 0.05) or by *L*. *acidophilus* supplementation (*P* > 0.05). To determine the similarities between pairs of microbial communities (β-diversity, Fig 1B), principal component analysis (PCA) was performed. Due to the high inter-individual variation, no distinguishable clustering of the ileal samples by dietary and/or infectious interventions was evident.

**Table 1:** The α diversity of ileal samples.

Items	Observed species	Shannon	Simpson	Chao1	ACE
CTL	343.83	3.49	0.73	414.33	417.82
LA	308.83	2.48	0.60	386.37	400.33
CLG	284.83	3.36	0.74	334.64	337.75
LACLG	281.67	2.92	0.70	343.70	344.67
SEM	10.297	0.167	0.032	13.288	13.044
Main effects					
*L*. *acidophilus*					
No addition	314.33	3.42	0.73	374.49	377.79
Addition	295.25	2.70	0.65	365.04	372.50
*C*. *perfringens* challenge					
Negative	326.33	2.98	0.67	400.35	409.08
Positive	283.25	3.14	0.72	339.17	341.21
*P*-value					
*C*. *perfringens* challenge	0.035	0.606	0.431	0.022	0.009
*L*. *acidophilus*	0.329	0.030	0.231	0.705	0.823
*C*. *perfringens* challenge × *L*. *acidophilus*	0.414	0.369	0.525	0.460	0.606

OTUs were taxonomically categorized via the LCA classifier trained on the Silva database with a minimal confidence score of 0.8. The relative abundance of OTUs was analyzed at different ranking levels: from phyla to genera. At the phylum level, the ileal microbiota was mainly composed of *Firmicutes* (>68%), followed by *Proteobacteria*, *Bacteroidetes*, and *Cyanobacteria*, whereas the probiotic *L*. *acidophilus* supplementation increased the relative abundance of *Firmicutes* in the ileal microbiota (S1A Fig). The *C*. *perfringens* infection numerically decreased relative abundance of the *Peptostreptococcaceae* family, and numerically increased the relative abundance of the *Enterobacteriaceae* family which was mainly composed of *Escherichia-Shigella* genera. In contrast, the probiotic *L*. *acidophilus* numerically increased the relative abundance of *Lactobacillaceae*, which was mainly composed of the *Lactobacillus* genus, and numerically decreased the relative abundance of *Escherichia-Shigella* genera (S1B and S1C Fig). Three taxonomic biomarkers (LDA score > 2) in the ileal microbial communities of groups CTL and CLG were identified by LEfSe method (Fig 1C). The relative abundance of *Lachnospiraceae* (Fig 1D) and *Ruminococcaceae* (Fig 1E) was significantly (LDA score > 2) higher in the CTL group than in other treatment groups; however, the relative abundance of *Gammaproteobacteria* was significantly (LDA score > 2) higher in the CLG group than in the treatment groups (Fig 1F).

### Effects of the *C*. *perfringens* challenge and *L*. *acidophilus* supplementation on the cecal microbiota

**Fig 2:**
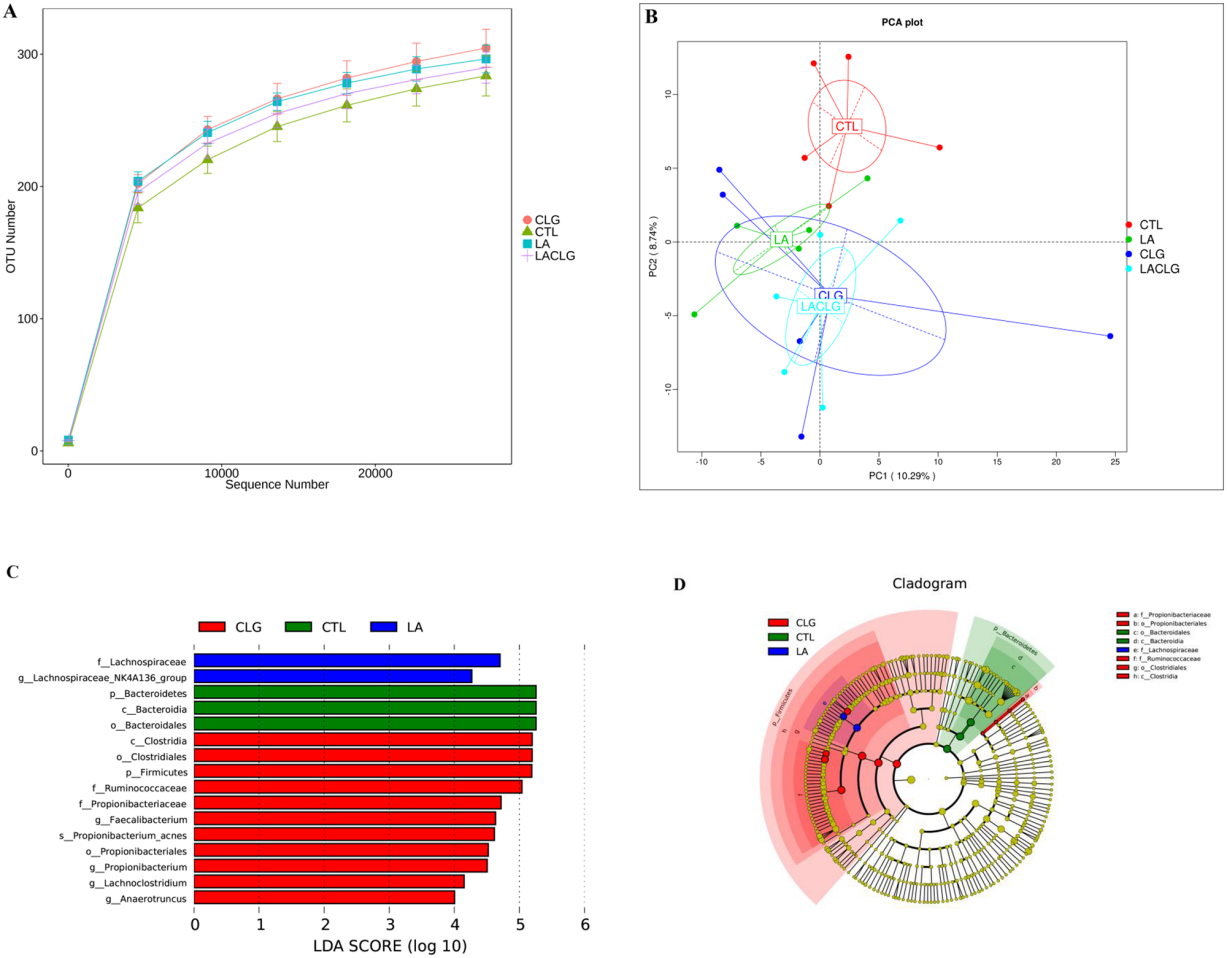
Diversity and composition of cecal microbiota. There were significant interaction effects of *L*. *acidophilus* and the infectious challenge on the number of observed OTUs (**A**). The PCA plot shows separation of bacterial communities between the CTL group and LA group (*R* = 0.42, *P* = 0.024), between the CTL group and CLG group (*R* = 0.404, *P* = 0.027), and between the CTL group and LACLG group (*R* = 0.50, *P* = 0.012) (**B**). Key phylotypes in the cecum responding to treatments were identified by the LEfSe algorithm (**C**). The circular cladogram (**D**) shows the taxa that are significantly associated with treatments.

Rarefaction curves of the observed OTUs in the cecum indicated that there were comparable numbers of OTUs among treatment groups (Fig 2A and Table 2). The number of observed species, and Shannon and Simpson indices of the CTL group were the lowest among the four groups (*P* < 0.05), and the *C*. *perfringens* challenge decreased the Chao1 index of the cecal bacterial community (*P* < 0.05). This community’s structure (β-diversity) in broiler chickens was compared among the four treatment groups by PCA. Principal component axis 1 explained 10.29% of the variation in the bacterial diversity, whereas the second axis explained 8.74% (Fig 2B).

**Table 2:** The α diversity of cecal samples.

Items	Observed species	Shannon	Simpson	Chao1	ACE
CTL	316.50^b^	4.67^b^	0.86^b^	370.00	374.66
LA	337.33^a^	5.54^a^	0.94^a^	365.88	359.96
CLG	332.67^a^	5.31^a^	0.91^a^	384.72	389.04
LACLG	331.17^a^	5.22^a^	0.91^a^	385.41	382.39
SEM	2.150	0.094	0.009	4.060	5.985
Main effects					
*C*. *perfringens* challenge					
Negative	326.92	5.11	0.90	367.94	367.31
Positive	331.92	5.27	0.91	385.07	385.71
*L*. *acidophilus*					
No addition	324.58	4.99	0.89	377.36	381.85
Addition	334.25	5.38	0.93	375.64	371.17
*P*-value					
*C*. *perfringens* challenge	0.139	0.295	0.392	0.039	0.135
*L*. *acidophilus*	0.007	0.015	0.019	0.829	0.379
*C*. *perfringens* challenge × *L*. *acidophilus*	0.002	0.003	0.013	0.762	0.738

With the Silva classifier, > 99.60% of the total sequence reads were assigned to the bacterial phyla. At the phylum level, the cecal microbiota was dominated by *Firmicutes* (> 45%), followed by *Bacteroidetes*, *Proteobacteria* and *Tenericutes* (S2A Fig). The *C*. *perfringens* infection increased the relative abundance of the *Escherichia-Shigella* genus (S2B Fig) in the cecal microbiota. LEfSe detected a marked increase (LDA score > 4) in the relative abundance of the *Clostridiales* family in the chicks of the CLG group compared with other groups (Fig 2C and 2D).

### Comparison of the microbiota between the ileum and cecum

**Fig 3:**
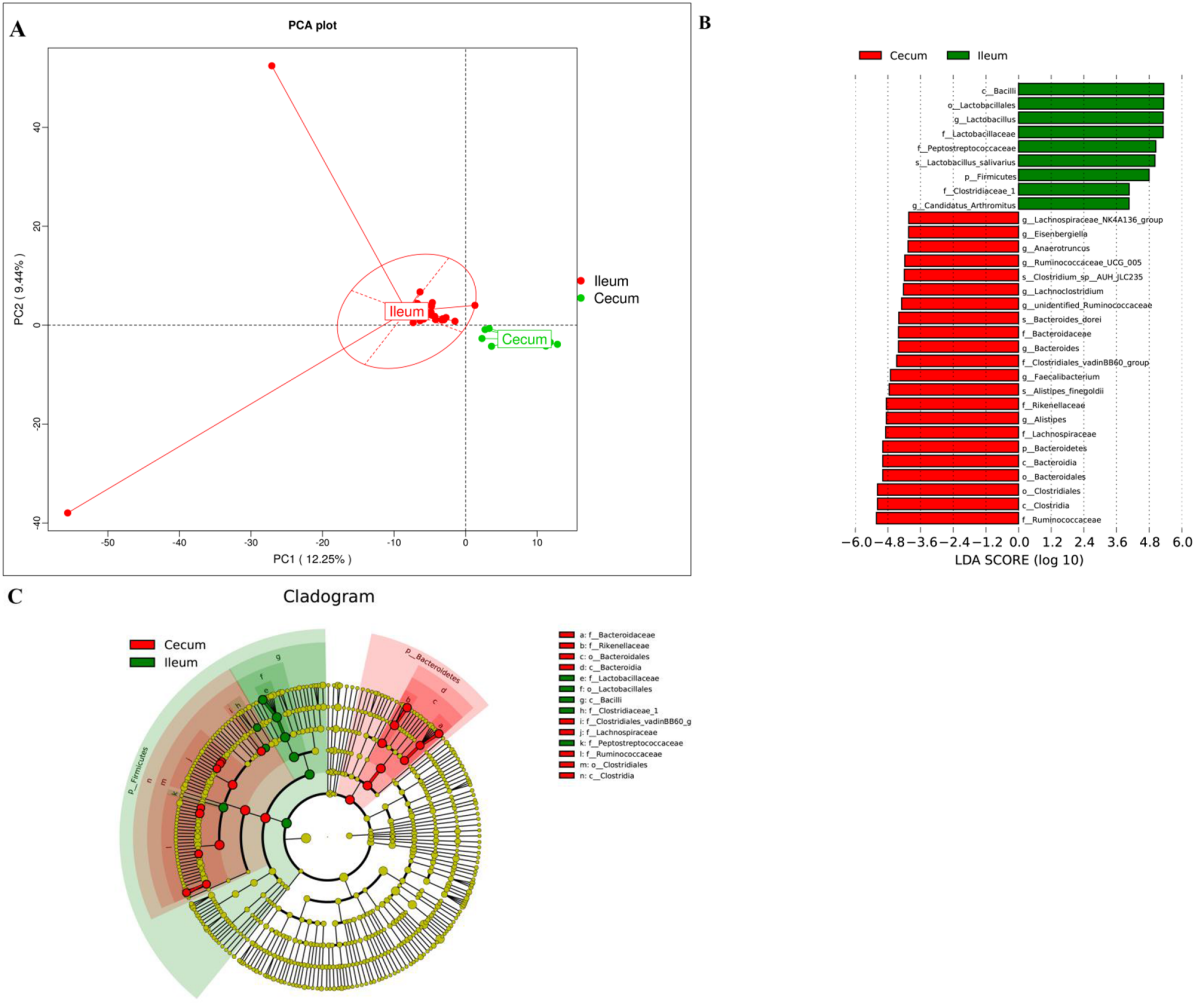
A comparison of diversity and composition between ileal and cecal microbiotas. The PCA plot (A) shows that the bacterial profile differed strongly by sampling site (*R* = 0.9138, *P* = 0.001). Taxa significantly associated with communities of ileum *versus* cecum were identified by the LEfSe algorithm (B) and are shown in the circular cladogram (C).

The α diversity indices of Shannon and Simpson were higher (*P* < 0.05) in the cecal samples than in the corresponding ileal samples (Table 3), indicating that the cecal microbiota was more diverse than the ileal microbiota. PCA of OTUs from the microbiotas in the ileum and cecum (Fig 3A) also revealed that the bacterial community structure differed significantly by sampling site (ANOSIM: *R* = 0.9138, *P* = 0.001). According to the LEfSe results, there were 31 differentially abundant bacterial clades at all taxonomic levels (LDA score > 2.0) between the ileal and cecal microbiotas (Fig 3B). *Peptostreptococcaceae*, *Lactobacillaceae* and *Enterobacteriaceae* were the dominant families in the ileum while the cecum was inhabited mostly by families *Ruminococcaceae*, *Rikenellaceae* and *Lachnospiraceae* (S3A Fig). The most dominant genus in the ileum was *Lactobacillus* accounting for more than 43% of all the observed sequence reads (S3B Fig).

**Table 3:** A comparison of α-diversity between ileal and cecal samples.

Items	Observed species	Shannon	Simpson	Chao1	ACE
Ileum	300.75	2.90	0.67	370.16	370.56
Cecum	306.75	5.14	0.91	360.17	355.30
SEM	6.097	0.209	0.027	8.398	7.944
*P* value	0.629	< 0.001	< 0.001	0.559	0.343

### The concentration of lactate and SCFAs in the ileum and cecum

The lactate and SCFA concentrations in the ileum (Table 4) and cecum (Table 5) were measured to test whether the observed microbial changes due to the infectious challenge and *L*. *acidophilus* supplementation affected intestinal function. In the ileum, the *C*. *perfringens* challenge tended to increase the lactate concentration (*P* = 0.061), and decreased the concentrations of formate (*P* < 0.05), acetate (*P* < 0.05) and propionate (*P* < 0.05), whereas *L*. *acidophilus* addition to the diet increased the concentration of lactate (*P* < 0.05) and butyrate, and decreased the concentrations of formate (*P* < 0.05) and propionate (*P* < 0.05). In contrast, isobutyrate, isovalerate and valerate were not detected in the ileum.

**Table 4:** Lactate and SCFA concentrations in the ileum.

Items^1^	Lactic acid	Formate	Acetate	Propionate	Butyrate
CTL	1347.41	111.14	173.45	5.79	0.73
LA	4205.59	79.46	92.76	3.26	3.18
CLG	2450.45	72.52	63.76	3.72	2.12
LACLG	7453.46	62.57	28.54	2.62	3.72
SEM	702.105	4.610	18.719	0.326	0.390
Main effects					
*C*. *perfringens* challenge					
Negative	2776.50	95.30	133.10	4.52	1.96
Positive	4951.95	67.54	46.15	3.17	2.92
*L*. *acidophilus*					
No addition	1898.93	91.83	118.60	4.57	1.42
Addition	5829.53	71.01	60.65	2.94	3.45
*P* value					
*C*. *perfringens* challenge	0.061	< 0.001	0.014	0.008	0.161
*L*. *acidophilus*	0.002	0.001	0.087	0.001	0.006
*C*. *perfringens* challenge × *L*. *acidophilus*	0.339	0.068	0.489	0.133	0.529

**Table 5:** Lactate and SCFA concentrations in the cecum.

Items^1^	Lactic acid	Formate	Acetate	Propionate	Isobutyrate	Butyrate	Isovalerate	Valerate
CTL	55.25	140.10	3476.65	340.32	28.31	930.79	17.53	59.73
LA	62.22	164.61	3310.78	399.83	24.87	1337.39	16.78	75.29
CLG	29.04	169.42	3384.29	230.14	15.86	1128.09	5.95	47.73
LACLG	140.63	126.69	3344.76	291.89	11.08	1052.34	14.78	70.51
SEM	15.135	9.022	120.136	36.174	2.891	72.924	1.985	4.648
Main effects								
*C*. *perfringens* challenge								
Negative	58.74	152.36	3398.72	370.07	26.59	1134.09	17.16	67.51
Positive	84.84	148.06	3364.52	261.01	13.47	1090.21	10.36	59.12
*L*. *acidophilus*								
No addition	42.14	154.76	3435.47	285.23	22.09	1029.44	11.74	53.73
Addition	101.43	145.65	3327.77	345.86	17.97	1194.86	15.78	72.90
*P* value								
*C*. *perfringens* challenge	0.338	0.810	0.895	0.148	0.024	0.760	0.083	0.350
*L*. *acidophilus*	0.037	0.612	0.678	0.412	0.454	0.257	0.290	0.041
*C*. *perfringens* challenge × *L*. *acidophilus*	0.063	0.072	0.793	0.988	0.903	0.104	0.212	0.685

In the cecum, the *C*. *perfringens* challenge significantly decreased the isobutyrate concentration (*P* < 0.05), and tended to decrease the isovelerate concentration (*P* = 0.081). *L*. *acidophilus* supplementation significantly increased the concentration of lactate (*P* < 0.05) and valerate (*P* < 0.05).

The correlation analyses indicated that the relative abundance of the ileal *Lactobacillus* genus positively correlated with ileal lactate production (*R* = 0.728, *P* < 0.001), whereas relative abundance of the *Propionibacterium* genus in the ileum of broiler chickens correlated with cecal valerate concentration negatively (*R* = -0.443, *P* < 0.05).

## Discussion

The gut microbiota is of great importance to host health and production. Many studies have revealed that the gut microbiota can provide the host with vitamin E, SCFAs, and other benefits [16]. Furthermore, gut microbiota is useful for the development of the host immune system [17]. Any alteration in the intestinal microbiota may have functional consequences for the health of the host [18]. Nowadays, analysis of 16S rRNA gene amplicons is a popular method for identifying the functional diversity [19] and variability [20] of the microbiome in the intestine of broiler chickens. In the present study, we used high-throughput sequencing of the V3-V4 region of the 16S rRNA gene to monitor the ileal and cecal microbiota of individual unchallenged or challenged broiler chickens fed a diet without or with *L*. *acidophilus* supplementation in a battery cage trial.

In our previous study, impaired growth performance of broilers, increased mortality and intestinal lesion scores after a *C*. *perfringens* challenge indicated that an experimental NE model was successfully established, and the dietary supplementation with *L*. *acidophilus* improved the growth performance and intestinal health of broilers (manuscript under review). Our current results indicate that the *C*. *perfringens* challenge and *L*. *acidophilus* supplementation modified the proportion of specific OTUs and these changes were associated with ileal lactate production.

In general, a more diverse microbial community shows stronger homeostasis of the intestinal microbial community and resistance to pathogens [21]. Increased gut microbial diversity is healthier in the elderly [22]. Another published study showed that the gut microbial diversity of athletes is higher than that of controls [23]. Low diversity of microorganisms is associated with a plethora of diseases, such as inflammatory bowel diseases [24, 25]. One study revealed that a *C*. *perfringens* challenge numerically decreases the α-diversity index of the small intestinal microbial community [18], as proved in our study. Nevertheless, other studies indicate that a *C*. *perfringens* challenge in the absence of predisposing factors does not cause significant changes in either α or β diversity of the cecal microbiota [26, 27]. The discrepancy in findings may be due to difference in *C*. *perfringens* strains and dissimilar diet types. Here, *L*. *acidophilus* supplementation of the diet decreased the ileal Shannon index, namely decreased the α-diversity of the ileal microbiota; this result can be explained as follows: *L*. *acidophilus* dietary supplementation made *L*. *acidophilus* the dominant bacterium in the ileum.

Our results on β-diversity revealed that the ileal microbial communities of four treatment groups were not separated clearly; this finding impliesthat chickens shared a core set of bacterial taxa in the ileum regardless of treatments. Plenty of studies indicate that *Firmicutes* rank first at the phylum level in terms of relative abundance in the intestinal bacterial community of broiler chickens [28–30]. In the present study, the *C*. *perfringens* challenge decreased the relative abundance of *Firmicutes* and increased the relative abundance of *Proteobacteria*. In the ileum, enrichment of the phylum *Firmicutes* and a reduction in the phylum *Proteobacteria* were observed after the probiotic *L*. *acidophilus* supplementation. We identified a class of bacteria that is associated with the CLG treatment, namely, *Gammaproteobacteria*, which belong to the phylum *Proteobacteria*. *Firmicutes* have a positive correlation with high energy efficiency, and the *Firmicutes*-to-*Bacteroides* ratio affects the amount of energy extracted from the diet [31–34]. Consequently, these parameters are directly related to the growth performance of broilers [35, 36]. *Proteobacteria* include many pathogens, such as subgroup *Salmonella*, *E*. *coli*, and *Shigella*, which can colonize both humans and chickens [37] and may trigger some specific disease. Therefore, the increase in *Firmicutes* and a reduction in *Proteobacteria* in the ileum may be associated with the improvement of growth performance and intestinal health of broilers fed with *L*. *acidophilus*. The ileal microbiota in this study was mainly composed of the genera *Lactobacillus* and *Escherichia-Shigella*, a finding that is consistent with other studies [29, 38]. In cecal samples here, most of the OTUs belonged to genera *Alistipes*, *Bacteroides*, *Faecalibacterium*, and *Escherichia-Shigella*.

*Lactobacillus*, as a typical probiotic bacterium, promotes the homeostasis of immune cells and intestinal health of the host [39, 40]. After a *C*. *perfringens*-challenge, *Lactobacillus* is suppressed at the early stage [18]. Nonetheless, other studies have shown that the number of is increased by an infectious challenge [9, 41], as confirmed in our present study. Some *Lactobacillus* species, such as *L*. *aviarius*, are suppressed by *C*. *perfringens* infection in the ileum of broilers [42]. The *Lactobacillus* genus was not dominant in the cecal microbiota in this study and was not affected by the infection. The *Enterobacteriaceae* family mainly consists of the *Escherichia-Shigella* genus, whose relative abundance was increased numerically by the infection in the present study, was and it represents major pathogenic microbes coexisting with NE in the intestine [43]. The probiotic *L*. *acidophilus* supplementation numerically decreased the relative abundance of *Escherichia-Shigella* genus both in the ileum and cecum and thereby further improved intestinal health.

SCFAs are defined as the class of fatty acids with fewer than six carbons, including formic acid (C1), acetic acid (C2), propionic acid (C3), butyric acid (C4) and valeric acid (C5) [44]. SCFAs are necessary for intestinal functionality and integrity [45], energy intake of enterocytes and cellular proliferation and differentiation within the intestinal mucosa [46]. These by-products are beneficial to broiler energy metabolism and contribute to lower pH of the broiler gut environment; this acidic pH may inhibit the overgrowth of intestinal pathogens [47]. Nutrients in the diets can be fermented by the gut microbiota, accompanied by the production of SCFAs and lactate [48]. The probiotic *L*. *acidophilus* supplementation increased the relative abundance of *Lactobacillus* in the intestine but might have suppressed some SCFA-producing bacteria, such as *Faecalibacterium* spp. (S1C Fig). Our results indicate that the ileal microbiota of chickens in the control group contained a higher proportion of butyrate-producing families *Ruminococcaceae* and *Lachnospiraceae*, which might have been suppressed in other experimental groups by the *C*. *perfringens* challenge or *L*. *acidophilus* supplementation. The lactate produced by *Lactobacillus* species, can be converted to butyrate, propionate, and acetate [49–51]. This notion was substantiated in the current study by the increased butyrate concentration in the ileum after *L*. *acidophilus* supplementation in comparison with control groups. Some SCFAs, mainly butyrate, are metabolized by the intestinal epithelial cells [52], and the others enter the diverse carbohydrate and lipid metabolic routes: propionate will mainly participate in gluconeogenesis while acetate and butyrate will be mostly processed in lipid biosynthesis [53]. Acetate has been proved to be the key *Bifidobacteria*-generated inhibitor of enteropathogens’ growth [54]. Butyrate nourishes the enterocytes, increases intestinal mucin production, which may change the bacterial adhesion [55] and improve tight-junction integrity [56], takes part in cellular differentiation and proliferation within the intestinal mucosa [46], and can reduce an inflammatory response as an anti-inflammatory effector [57]. *C*. *perfringens* infection damages the intestinal mucosa of broilers [9, 58] and disrupts homeostasis of the intestinal microbiota [18]. Another study revealed that a *C*. *perfringens* challenge in the absence of predisposing factors does not cause significant changes in concentrations of SCFAs [26]. In the present study, however, the infection decreased the ileal concentration of formate, acetate, and propionate and cecal isobutyrate concentration; these alterations might have resulted frominhibition of the growth of a few bacterial taxa that can produce SCFAs. The probiotic *L*. *acidophilus* supplementation increased the relative abundance of *Lactobacillus* and concentrations of lactate and SCFAs in the intestine of broilers. Our results showed that the ileal concentrations of lactate and butyric acid and cecal concentrations of lactate and valerate increased with the *L*. *acidophilus* supplementation, and consequently alleviated the damage caused by *C*. *perfringens* infection and restored the disrupted gut microbiota.

## Conclusion

We found that dietary supplementation with probiotic *L*. *acidophilus* increases the relative abundance of beneficial bacteria and decreases the relative abundance of pathogens in the intestine of broilers challenged by *C*. *perfringens* infection. Increased population of *Lactobacillus*, elevated concentrations of lactate and butyrate, and decreased relative abundance of *Escherichia*-*Shigella* may promote intestinal health and contribute to the recovery of an intestinal microbial community disrupted by *C*. *perfringens* infection.

## Supporting information

S1 Fig The top 10 phyla (A), families (B) and genera (C) in the ileal microbial community on day 21. (TIF)

S2 Fig The top 10 phyla (A) and genera (B) in the cecal microbial community on day 21. (TIF)

S3 Fig The top 10 families (A) and genera (B) in the ileal and cecal microbial communities on day 21. (TIF)

S1 Table Composition of the basal diet (as-fed basis). (DOCX)
