# Supplementary figures and images for: Effects of Lactobacillus acidophilus on gut microbiota composition in broilers challenged with Clostridium perfringens
Source: PLoS One. 2017 Nov 30;12(11):e0188634. doi: 10.1371/journal.pone.0188634 (PMC5708699; doi:10.1371/journal.pone.0188634)

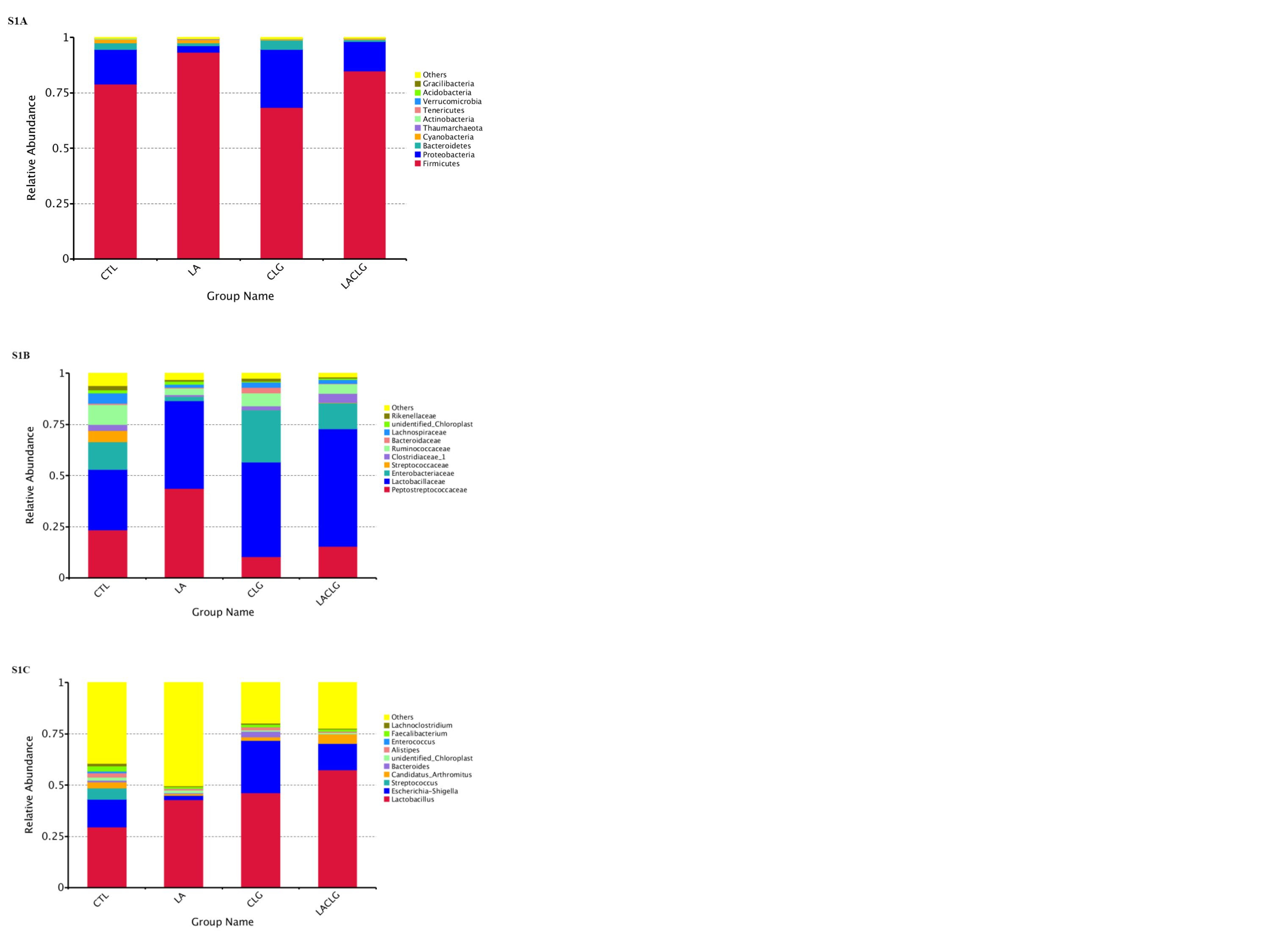

Supplement: S1 Fig — (TIF) [file pone.0188634.s001.tif]

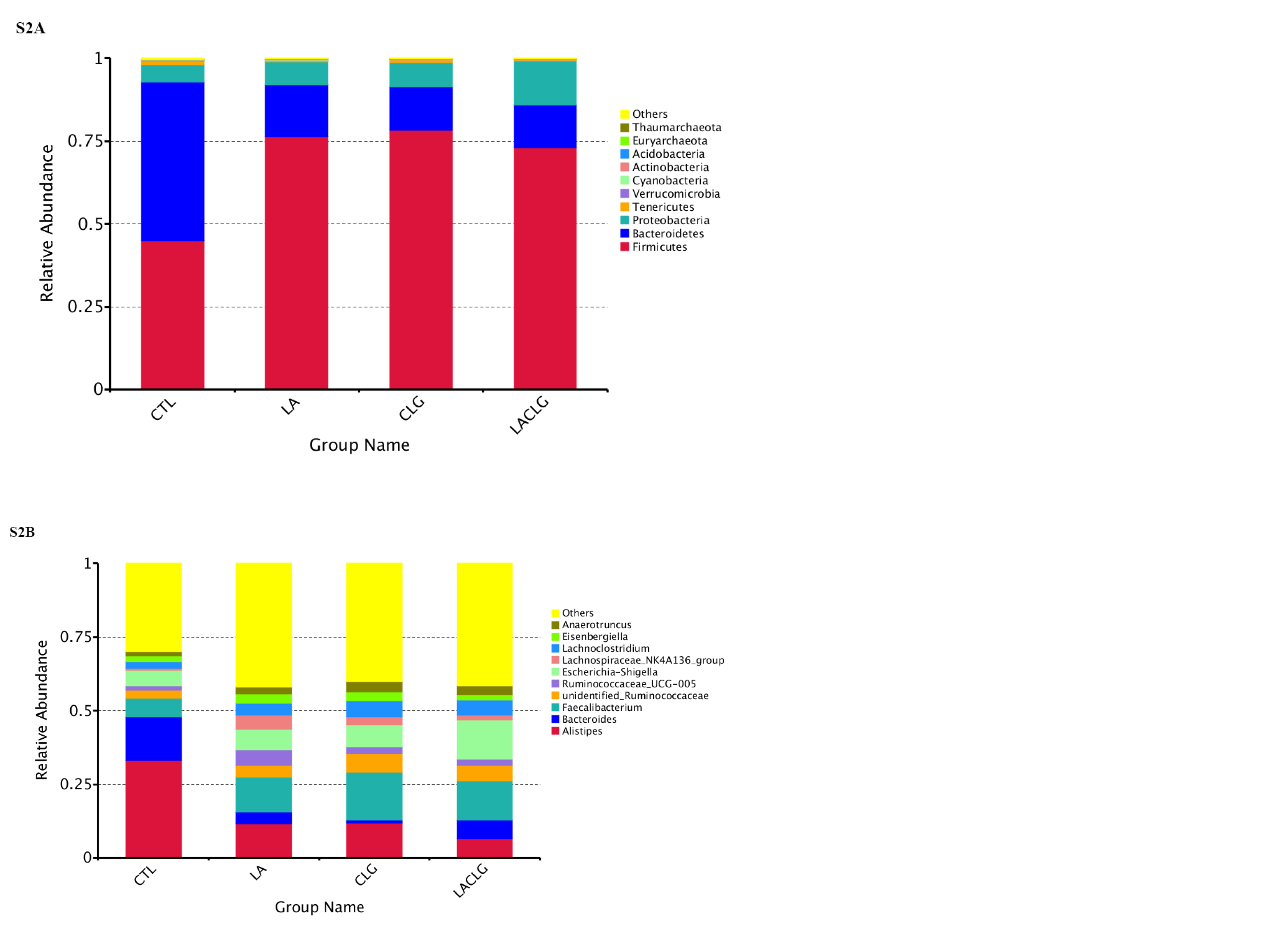

Supplement: S2 Fig — (TIF) [file pone.0188634.s002.tif]

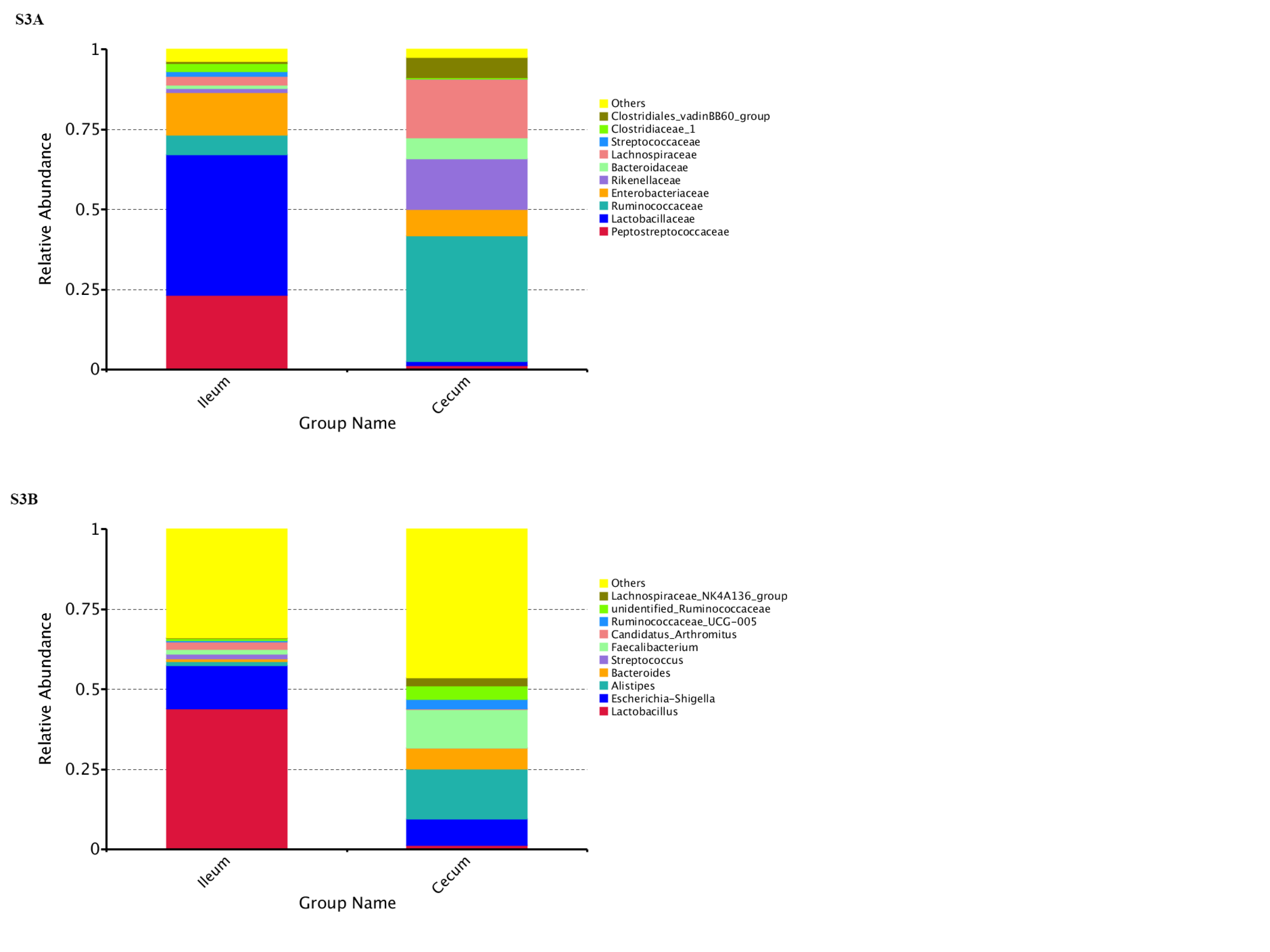

Supplement: S3 Fig — (TIF) [file pone.0188634.s003.tif]
