# Supplementary material for: Effects of Lactobacillus acidophilus on gut microbiota composition in broilers challenged with Clostridium perfringens
Source: PLoS One. 2017 Nov 30;12(11):e0188634. doi: 10.1371/journal.pone.0188634 (PMC5708699; doi:10.1371/journal.pone.0188634)
Supplement: S1 Table — (DOCX) [file pone.0188634.s004.docx]

**S1 Table. Composition of basal diet (as-fed basis).**

| Item | Composition |
| --- | --- |
| Ingredients, % |  |
| Corn | 51.38 |
| Soybean oil | 3.75 |
| Soybean meal | 40.71 |
| CaHPO_3_.2H_2_O | 1.86 |
| Limestone | 1.24 |
| NaCl | 0.35 |
| *DL*-Met | 0.20 |
| Vitamine premix^1^ | 0.03 |
| Trace mineral premix^2^ | 0.20 |
| 50% Choline chloride | 0.25 |
| Antioxidant | 0.03 |
| Total | 100 |
| Nutrient level^3^ |  |
| Metabolizable energy, MJ/Kg | 12.31 |
| Crude protein, % | 22.00 |
| Lys, % | 1.21 |
| Met, % | 0.52 |
| Ca, % | 1.00 |
| Available phosphorus, % | 0.45 |

^1^Vitamin premix (1 kg) contained: vitamin A, 50 MIU; vitamin D_3_, 12 MIU; vitamin K_3_, 10 g; vitamin B_1_, 10 g; vitamin B_2_, 32 g; vitamin B_12_, 0.1 g; vitamin E, 0.2 MIU; biotin, 0.5 g; folic acid, 5 g; pantothenic acid, 50 g; niacin, 150 g.

^2^Trace mineral premix (1 kg) contained: copper, 4 g; zinc, 90 g; iron, 38 g; manganese, 46.48 g; selenium, 0.1 g; iodine, 0.16 g; cobalt, 0.25 g.

^3^Calculated value based on the analyzed data for the experimental diets.
